# Asymmetric dynamic linkage between consumer sentiment, inflation expectations, and international energy prices: Evidence from time-frequency wavelet and nonlinear analysis

**DOI:** 10.1371/journal.pone.0308097

**Published:** 2024-09-03

**Authors:** Lianlian Fu, Dongyu Yuan, Jiamin Teng

**Affiliations:** School of Computer and Information Engineering, Jiangxi Agricultural University, Nanchang, China; Roma Tre University: Universita degli Studi Roma Tre, ITALY

## Abstract

This study investigates the relationship between consumer sentiment (CONS), inflation expectations (INEX) and international energy prices, drawing on principles from behavioral. We focus on Brent crude oil price and Henry Hub natural gas prices as key indicators of energy market dynamics. Based on the monthly data from January 2003 to March 2023, three wavelet methods are applied to examine the time-frequency linkage, while the nonlinear distributed lag model (NARDL) is used to verify the asymmetric impact of two factors on energy prices. The results highlight a substantial connection between consumer sentiment, inflation expectations and international energy prices, with the former in the short term and the latter in the medium to long term. Especially, these correlations are particularly pronounced during the financial crisis and global health emergencies, such as the COVID-19 epidemic. Furthermore, we detect short-term asymmetric effects of consumer sentiment and inflation expectations on Brent crude oil price, with the negative shocks dominating. The positive effects of these factors on oil prices contribute to observed long-term asymmetry. In contrast, inflation expectations have short-term and long-run asymmetric effects on natural gas price, and both are dominated by reverse shocks, while the impact of consumer sentiment on natural gas prices appears to be less asymmetric. This study could enrich current theories on the interaction between the international energy market and serve as a supplement to current literature.

## 1. Introduction

In recent years, the international energy sector has experienced significant price volatility, with crude oil, often referred to as the “the king of commodities”, playing a pivotal role in global financial markets. Its fluctuations of price have been a persistent concern for various industrial sectors [1]. Hit by COVID-19 in 2019, oil price fell to 40 dollars per barrel. Since 2022, the Russian-Ukrainian conflict triggered an energy crisis, and a variety of over-anticipated factors have exacerbated the sharp fluctuations of energy markets represented by crude oil [2]. The crude oil price approached the historical extreme value last seen before the financial crisis in 2008. Similarly, as the fossil fuel with the lowest carbon emissions, the stable development of natural gas is crucial for reducing carbon emissions [3]. The price of natural gas has been relatively stable throughout history, except for 2006 and 2008. As the representative energy sources of the international energy market, crude oil and natural gas have been selected as research objects by many scholars.

It is widely recognized that volatility in energy prices has an impact on many financial markets such as agricultural markets [4], food markets [5], stock markets [6] and carbon markets [7], contributing to global economic uncertainty. Furthermore, various macroeconomic factors such as economic policy uncertainty [8] have been proven as the main factors that influence the volatility of energy prices. However, the subjective perspective of the consumers, particularly their psychological expectations, has been less investigated in relation to energy prices. It is plausible that changes in psychological sentiment and expectations, etc., could affect the volatility of distinct types of energy prices by influencing consumer decision-making and behavior. Although this category of indicators has been selected by some scholars as factors influencing consumption behavior [9, 10], there is a scarcity of literature linking these factors directly to energy prices. Hence, it is of great practical significance to identify and analyze the dynamic linkage between potential factors that affect energy prices.

In this study, we choose consumer sentiment (CONS) and inflation expectations (INEX) as potential factors affecting energy prices for empirical analysis. Consumer sentiment, reflecting consumers’ comprehensive judgements on employment, income, prices, and interest rates, is a subjective experience of whether an objective thing meets one’s needs and has long been recognized as a major driving force of economic activity [11]. It affects consumer behavior, which in turn affects consumer demand and the profitability of the sales side, impacting the pricing strategies. If consumers are optimistic about the future economic outlook, they may be more inclined to purchase energy-intensive goods and services, such as cars and travel products, increasing energy demand and prices. Conversely, pessimistic consumer sentiment could lead to reduced purchases, resulting in a decrease in energy demand and prices [12]. Consumer sentiment will be affected by shocks from external events, with one research finding that changes in consumer sentiment dramatically affected their consumption behavior after a shock from COVID-19, which lasted for a year [13]. A handful of scholars has already proved the correlation between sentiment indicators and several types of prices. For example, in the case of the agricultural market, COVID-19 has a serious influence on the stability of public sentiment, which triggered panic and certain irrational purchasing behaviors, and further generated a negative impact on the prices of agricultural products [14]. Zhao et al. [15] investigated the heterogeneity of the influence of online investor sentiment on WTI crude oil price before and after the COVID-19 outbreak by constructing a SAVAR model.

Inflation expectations, which are psychological anticipations of future events rather than actual inflation, can also influence consumer spending and investment behavior. Inflation itself will give rise to fluctuations in prices, exchange rates, etc. [16], and individuals will anticipate in advance to guard against impending inflation and will then engage in a range of behaviors such as spending in advance and hoarding goods, which will exacerbate inflation, thus bringing about increased market instability. Strong inflation expectations can lead to an inflationary spiral, affecting economic and social stability. Consumer expectations of inflation, which are at the heart of macroeconomic modelling and monetary policy, are having an increasingly visible impact on economic outcomes [17]. Similarly, there exists a clear moderating role for inflation expectations in the transmission of exchange rates. The exchange rate will be less unstable when the market and consumers have advanced expectations of high inflation in the future [18]. A slice of studies has already confirmed the significant impact of oil and other energy price changes on real inflation [19, 20]. As a leading indicator of inflation, inflation expectations have become a focal point of academic attention at present [21].

As research deepens, scholars find that the linkage between energy prices and various indicators is not a simple linear causality but a more complex dynamic correlation involving time and frequency. Wavelet analysis, a promising methodology for exploring the time-frequency patterns of non-stationary time series, is increasingly applied in the financial field. For instance, Kristoufek [22] utilized the continuous wavelets framework to explore the main drivers of the Bitcoin price. By the multi-scale wavelet approach, Bekiros et al. [23] analyzed the co-movements between stock markets and gold markets of BRICS countries and proved the strong dependence structure. This paper employs cross-wavelet, partial and multiple wavelet coherence techniques as the primary methods by considering various types of wavelet analysis and related literature [24, 25]. In addition to the synergies and correlations in time and frequency, there are also evident asymmetric effects among financial variables. Specifically, prices respond differently to positive and negative shocks to numerous factors, and there is short-term and long-term heterogeneity. The nonlinear distributed lag model (NARDL) is an effective technique to investigate the asymmetric impact, which was proposed by Shin et al. [26]. These approaches above are different but complementary, and we conduct detailed explanations in the section of methodology.

Based on the above analysis, to probe the potential factors related to international energy prices, this paper first chooses two variables, consumer sentiment (CONS) and inflation expectations (INEX) defined by the University of Michigan to test their impact on energy prices; simultaneously, the spot price of crude oil in Brent and natural gas in Henry Hub are selected as the proxies for international energy prices. Then, cross-wavelet and two wavelet coherence techniques are applied to probe the dynamic correlation between consumer sentiment, inflation expectations and international energy prices; on this basis, the NARDL model is utilized to tap into the asymmetric impacts of consumer sentiment and inflation expectations on energy prices from long and short-term perspectives, respectively.

This paper contributes to the current literature surrounding international energy price in three aspects. Firstly, from a new perspective of behavioral economics, this study chooses two leading indicators of consumer sentiment and inflation expectations, which are subjective and psychological, to explore their impact on international energy prices. Secondly, our findings open a new insight into the potential correlation between influencing factors and price fluctuations both in time and frequency. To the best of our knowledge, most research focuses on the time domain dimension, with less attention paid to the frequency domain dimension. We employ three advanced wavelet techniques to analyze high-frequency(short-term) and low-frequency(long-term) correlation between consumer sentiment, inflation expectations, and two types of energy prices, which can assist the relevant departments in formulating more targeted policies to prevent risks. Thirdly, to further examine whether there exists an asymmetric effect of consumer sentiment and inflation expectations on energy prices, we apply the NARDL model to probe the nonlinear linkage between time series on the positive and negative shocks breaks through the limitation that most of the traditional econometric models could only be applied for linear relationships.

The remainder of this paper is organized as follows: Section 2 is the literature review, which provides a brief review of the existing literature on energy prices. Details on research methodology and data sources are illustrated in Section 3. Section 4 discusses the empirical results and then makes conclusions and policy recommendations in Section 5.

## 2. Literature review

Currently, a substantial body of literatures has examined the interconnections between various influencing factors and energy prices. In light of the escalating external uncertainty in recent years, the decision-making process of economic agents has been notably impacted, promoting an increasing number of energy market investors to seek portfolio diversification [27]. Numerous financial indicators have been utilized to investigate their relationship with energy prices. Economic policy uncertainty has been considered a crucial factor affecting the prices of diverse products. Feng et al. [28] identified a substantial positive correlation between economic policy uncertainty and stock price volatility within the energy sector. However, Reboredo et al. [29] found that market uncertainty conditions are not crucial enough in commodity prices, contradicting many studies related to policy uncertainty. Additionally, geopolitical risk [30] and exchange rates [31] are also important factors. During the COVID-19 pandemic and the global financial crisis, the volatility risk in financial markets tends to escalate [32]. The COVID-19 epidemic has been a major reference event in recent years, with Chen et al. [33] investigating the dynamic volatility contagion of the Baltic Dry Index (BDI), iron ore prices, and crude oil prices in the context of COVID-19. Their findings implies that the interdependencies among these variables are time-varying and exhibit time lags during significant crisis. Furthermore, growth and cyclical changes in the global economy also affect energy price volatility [34]. The selection of influencing factors has become more diverse, and in this regard, Zhao [35] constructed a GARCH-MIDAS model combining level and volatility effects, which is specifically from the commodity attributes, macroeconomic factors, alternative energy sources, and geopolitical events, to explore the several factors influencing crude oil price volatility.

Some scholars argue that leading indicators related to psychology and expectations are also crucial factors influencing economic activity. Daniel et al. [36] applied the PLS-SEM to confirm that emotions and subjective norms are the main factors influencing consumers to purchase fairtrade roses. Of course, these factors not only affect the behavior of the consumer, but also have complex linkages with prices themselves. As the research of Hensel et al. [37] confirms, that firms’ price expectations and decisions are affected by carbon pricing. Under the shock of various external emergencies, these linkages become increasingly evident. Bozma et al. [38] demonstrated that COVID-19 has disrupted the supply chain, and the intensification of inflation will further lead to increases in energy prices.

Wavelet analysis has been applied widely to explore dynamic linkage in time and frequency, and to describe the time-scale characteristics in energy, agricultural and other financial markets. Mastroeni et al. [39] exploited the wavelet analysis to explore the correlation between oil and food prices and its determinants in the domains of time and frequency. Similarly, there is also a time-frequency causality and time-varying co-movement between the US stock and commodity markets [40]. In terms of external macroeconomic factors, Cao et al. [41] utilized wavelet coherence to confirm the asymmetric impact of policy uncertainty on energy and food prices, especially in the context of major international events. Additionally, wavelet transforms have been applied to analyze the dynamic synergy between the investor sentiment index and energy price volatility risk [42], which holds certain reference value for the research object of this paper.

More current research has manifested that the impact of external influences on prices tends to be non-linear [43, 44], posing a challenge for traditional econometric models to estimate such relationships. Therefore, after identifying the correlation of variables utilizing wavelet techniques, it is indispensable to further inquiry the mechanism of asymmetric influence between variables in conjunction with other models. Various types of methods extended from VAR and linear regression, etc. are more often applied for the variables that are linearly related to each other [45]. The models based on copula and GARCH can capture nonlinear correlations among variables but tend to have too many parameters to estimate and are unable to describe the characteristics of different periods. The emergence of the NARDL model effectively addresses this issue, as it only requires a relatively small number of parameters to estimate and meticulously depicts the dynamic impacts in both short and long-run periods, verifying whether such impacts are asymmetric or not. Several scholars have chosen this model to confirm the existence of asymmetric effects. Kamaruddin et al. [46] examined the asymmetric transmission mechanism of coffee prices by the NARDL model. Waqar et al. [47] used this model to examine the transmission of oil prices to fares in the transport sector, determining the magnitude of positive and negative shocks of oil prices. Of course, the methodology is not limited to the study of price transmission. Rahman et al. [48] studied the asymmetric impact of FDI and public expenditure on population health, providing several policy recommendations to improve the life expectancy of the general population in Pakistan. Similarly, Sun et al. [49] looked into whether art serves as a hedge against economic policy uncertainty based on it, with significant implications for investors and collectors in the art market. In addition to the NARDL approach, other similar models such as QARDL [50], CS-ARDL [51] and BARDL [52] have been applied.

Synthesizing the aforementioned literature, our paper fills the void that exploring the dynamic linkage between consumer sentiment, inflation expectations, and international energy prices from time-frequency and asymmetric perspectives.

## 3. Methodology and data

### 3.1. Wavelet analysis

Wavelet analysis is a shared approach in the analysis of non-stationary time series and can capture the local frequency signature of an event hidden in long-time series data, making it an optimal tool for managing non-stationary series. Note that, the current literature on wavelets predominantly utilizes scale, period and horizon which correspond to the representation of wavelength on the vertical coordinate [53]. In this study, we have opted for the term “period” to align with standard terminology. The frequency, which is the inverse of the wavelength, signifies the varying relationships and movements between series. Movements at low frequencies reveal insights into the overall patterns of the time series, while high-frequency movements capture transient details. Therefore, low-frequency movements can be explained as long-term component, whereas high-frequency movements are related to short-term dynamics.

In this paper, the cross-wavelet transform is employed to measure the intensity of the "common cycle" shared between the series, indicating the degree to which they move in synchrony over time. Wavelet coherence, on the other hand, is applied to assess the consistency of the periodic "trend" between the series, revealing the stability of their relationship across different frequencies. These techniques allow for a nuanced understanding of the interactions between time series data, providing a comprehensive view of both short-term and long-term dynamics.

#### 3.1.1. Continuous wavelet transform and Morlet wavelet

Firstly, the continuous wavelet transform *W*_*x*_(*m*,*n*) is defined as:

$$W_{x}(m,n)=\int_{-\infty }^{\infty }x(t)\frac{1}{\sqrt{n}}\Psi (\frac{t-m}{N})dt$$ (1)

Among them, the mother wavelet *Ψ* is a continuous function both in the time and frequency domain and projected onto the time series *x*(*t*). Many wavelet functions can serve as the mother wavelet. In time series analysis, wavelet amplitudes need to be stationary and continuous, so choosing a nonorthogonal wavelet function is more appropriate. To obtain the amplitude and phase of the sequence, it is necessary to apply a complex-valued wavelet with unquantifiable component. Thus, we employ the Morlet wavelet in this paper, which is defined as:

$$\Psi (t)=\pi^{-\frac{1}{4}}e^{i\omega_{0}t}e^{-\frac{1}{2}t^{2}}$$ (2)

where *ω*_0_ is a dimensionless frequency and *t* is a dimensionless time. Following previous literature, *ω*_0_ is set to 6, which makes scale and periodic substitute for each other [54]. The value ensures that the scale is inversely related to frequency.

#### 3.1.2. Cross wavelet transform and phase difference

The cross-variation between the two-time series be expressed as:

$$W_{xy}(m,n)=W_{x}(m,n)W_{y}^{*}(m,n)$$ (3)

of which *W*_*x*_(*m*,*n*) and *W*_*y*_(*m*,*n*) denote the continuous wavelets transform of the two variables, respectively, and * denotes the composite covariance. The cross-wavelet power spectrum divides the intervals and highlights the high sensitivity attention in different time and frequency domains throughout the time series. WC coefficients adjusted by Torrence and Webster [55] are as follows:

$$R^{2}(m,n)=\frac{{\left|N(N^{-1}W_{xy}(m,n))\right|}^{2}}{N(N^{-1}{\left|W_{x}(m,n)\right|}^{2})N(N^{-1}{\left|W_{y}(m,n)\right|}^{2})}$$ (4)

where *N* is the smoothing operator and 0≤*R*^2^(*m*,*n*)≤1 is the squared WC coefficient that assumes the existence of a correlation between the variables. For instance, a value of the coefficient approaching 1 would indicate that an elevated level of correlation exists; on the contrary, it implies that there is no correlation. Additionally, the wavelet phase difference which represents the lead-lag relationship between variables can be defined as:

$$\varphi (m,n)={tan}^{-1}(\frac{ℶ(W_{xy}(m,n))}{ℵ(W_{xy}(m,n))})$$ (5)

Where ℵ and ℶ are real and imaginary components, respectively. According to the value of *φ*(*m*,*n*), we can distinguish the lead-lag relationship between variables. Specifically, there are several cases: (1) if *φ*(*m*,*n*) = 0, the series are in-phase (positive co-movement) and no lead(lag) relationship. The arrow will point to the right (→). (2) if $\varphi (m,n)\in (0,\frac{\pi }{2})$, the series are in-phase (positive co-movement) with *x* leading *y*. The arrow will point to right and up (↗). (3) if $\varphi (m,n)\in (\frac{\pi }{2},\pi )$, the series are out of phase (negative co-movement) with *y* leading *x*. The arrow will point to the right (↖). (4) if $\varphi (m,n)\in (-\frac{\pi }{2},0)$, the series are in-phase (positive co-movement) with *y* leading *x*. The arrow will point to the right (↘). (5) if $\varphi (m,n)\in (-\pi ,-\frac{\pi }{2})$, the series are out of phase (negative co-movement) with *x* leading *y*. The arrow will point to the right (↙).

#### 3.1.3. Partial wavelet coherence (PWC)

Partial wavelet coherence is well-suitable for correlation theory that enables the testing of simple time-varying correlations. The method can be applied in the detection of variables *x*_1_ and *x*_2_ coherence while eliminating the effects of variable *x*_3_ [24]. The partial correlation squared equation is expressed as:

$${RP}^{2}(x_{1},x_{2},x_{3})=\frac{{\left|R(x_{1},x_{2})-{R(x_{1},x_{2})}^{*}\right|}^{2}}{{\left[1-R(x_{1},x_{2})\right]}^{2}{\left[1-R(x_{3},x_{2})\right]}^{2}}$$ (6)

#### 3.1.4. Multiple wavelet coherence (MWC)

While ordinary wavelet coherence analysis can only be employed between two series, multiple wavelet coherence is a state-of-the-art method to study the correlation between multiple time series. This process focuses on the consistency of multiple predictor variables on the standard variable.


$${RM}^{2}(x_{1},x_{3},x_{2})=\frac{R^{2}(x_{1},x_{2})+R^{2}(x_{1},x_{3})-2R_{e}[R(x_{1},x_{2}).{R(x_{1},x_{3})}^{*}.{R(x_{3},x_{2})}^{*}]}{1-R^{2}(x_{3},x_{2})}$$ (7)

This equation represents the strength of the wavelet power of the two predictor variables *x*_2_ and *x*_3_ at different times and frequencies concerning the standard variable *x*_1_.

### 3.2. Non-linear distributed lag modelling (NARDL)

Shin et al. [26] proposed the NARDL model by considering asymmetric long-term regression, which is an extension of the ARDL model. Compared with other asymmetry research approaches, this method can be applied to small sample data, and is not disturbed by the endogeneity problem; the sequence does not need to satisfy all the same order single integer, it can be either 1st order single integer or 0th order single integer. Firstly, it is assumed:

$$y_{t}=\beta^{+}x_{t}+\beta^{-}x_{t}+u_{t}$$ (8)

$$\Delta x_{t}=v_{t}$$ (9)

The *x*_*t*_ is decomposed into $x_{t}^{+}$ and $x_{t}^{-}$ which represent the positive and negative changes:

$$x_{t}^{+}=\sum_{j=1}^{t}\Delta x_{j}^{+}=\sum_{j=1}^{t}\max\left(\Delta x_{j},0\right)$$ (10)

$$x_{t}^{-}=\sum_{j=1}^{t}\Delta x_{j}^{-}=\sum_{j=1}^{t}\min\left(\Delta x_{j},0\right)$$ (11)

The asymmetric cointegration model for the partial components is provided above. Smooth linear combinations of partial components are defined below:

$$z_{t}=\beta_{0}^{+}y_{t}^{+}+\beta_{0}^{-}y_{t}^{-}+\beta_{1}^{+}x_{t}^{+}+\beta_{1}^{-}x_{t}^{-}$$ (12)

If *z*_*t*_ is smooth, then *y*_*t*_ and *x*_*t*_ are “asymmetrically cointegrated”. Standard linear (symmetric) cointegration is a special case of (9) only if $\beta_{0}^{+}=\beta_{0}^{-}$ and $\beta_{1}^{+}=\beta_{1}^{-}$ can be obtained. Consider the case where the following restriction holds: $\beta_{0}^{+}=\beta_{0}^{-}=\beta_{0}$ in expression (9), which implies that $\beta^{+}=-\beta_{1}^{+}/\beta_{0}$ and $\beta^{-}=-\beta_{1}^{-}/\beta_{0}$.

Based on this, Shin et al. proposed the nonlinear ARDL(*p*,*q*) model:

$$y_{t}=\sum_{j=1}^{p}\varphi_{j}y_{t-j}+\sum_{j=0}^{q}\left(\theta_{j}^{{+}^{\prime }}x_{t-j}^{+}+\theta_{j}^{{-}^{\prime }}x_{t-j}^{-}\right)+\varepsilon_{t}$$ (13)

Where *x*_*t*_ is a *k*×1 multivariate regression quantity and *θ*_*j*_ is the autoregressive parameter. $\theta_{i}^{+}$ and $\theta_{j}^{-}$ are asymmetrically distributed lag parameters, and *ε*_*t*_ is an *i*.*i*.*d* process of zero-mean and constant-variance. Then *x*_*t*_ is decomposed near zero into $x_{t}^{+}$ and $x_{t}^{-}$, to distinguish the positive and negative changes in the growth rate of the *x*_*t*_ The error-corrected form is as follows:

$$\Delta y_{t}=\rho y_{t-1}+\theta^{{+}^{\prime }}x_{t-1}^{+}+\theta^{{-}^{\prime }}x_{t-1}^{-}+\sum_{j=1}^{p-1}\gamma_{j}\Delta y_{t-j}+\sum_{j=0}^{q-1}\left(\varphi_{j}^{{+}^{\prime }}\Delta x_{t-1}^{+}+\varphi_{j}^{{-}^{\prime }}\Delta x_{t-1}^{-}\right)$$ (14)

$$=\rho \xi_{t-1}+\sum_{j=1}^{p-1}\gamma_{j}\Delta y_{t-j}+\sum_{j=0}^{q-1}\left(\varphi_{j}^{{+}^{\prime }}\Delta x_{t-1}^{+}+\varphi_{j}^{{-}^{\prime }}\Delta x_{t-1}^{-}\right)$$ (15)

A simplified data generation process of Δ*x*_*t*_ was proposed to handle the non-zero relevance between regressions and residuals:

$$\Delta x_{t}=\sum_{j=1}^{q-1}\Lambda_{j}\Delta x_{t-j}+v_{t}$$ (16)

Where *v*_*t*_∼*iid*(0,*∑*_*v*_) and *∑*_*v*_ is a *k*×*k* positive definite covariance matrix of order. According to the conditional model, we denote *ε*_*t*_ as:

$$\varepsilon_{t}=\omega^{\prime }v_{t}+e_{t}=\omega^{\prime }(\Delta x_{t}-\sum_{j=1}^{q-1}\Lambda_{j}\Delta x_{t-j})+e_{t}$$ (17)

### 3.3. Data sources

To thoroughly investigate the dynamic linkage between consumer sentiment, inflation expectations and international energy prices, this paper chooses the spot price of Brent crude oil and natural gas as the proxy variables for international energy prices. These data are derived from the U.S. Energy Information Administration (EIA), a reputable provider of energy-related information. We utilize two leading indicators defined by the University of Michigan: Consumer Sentiment (CONS) and Inflation Expectations (INEX). These indicators are obtained from the Federal Reserve Economic Data (FRED) database. The interval of the above sample data is from January 2003 to March 2023, yielding a total of 243 monthly observations.

## 4. Results and discussion

### 4.1. Descriptive statistical analysis and smoothness test

**Fig 1:**
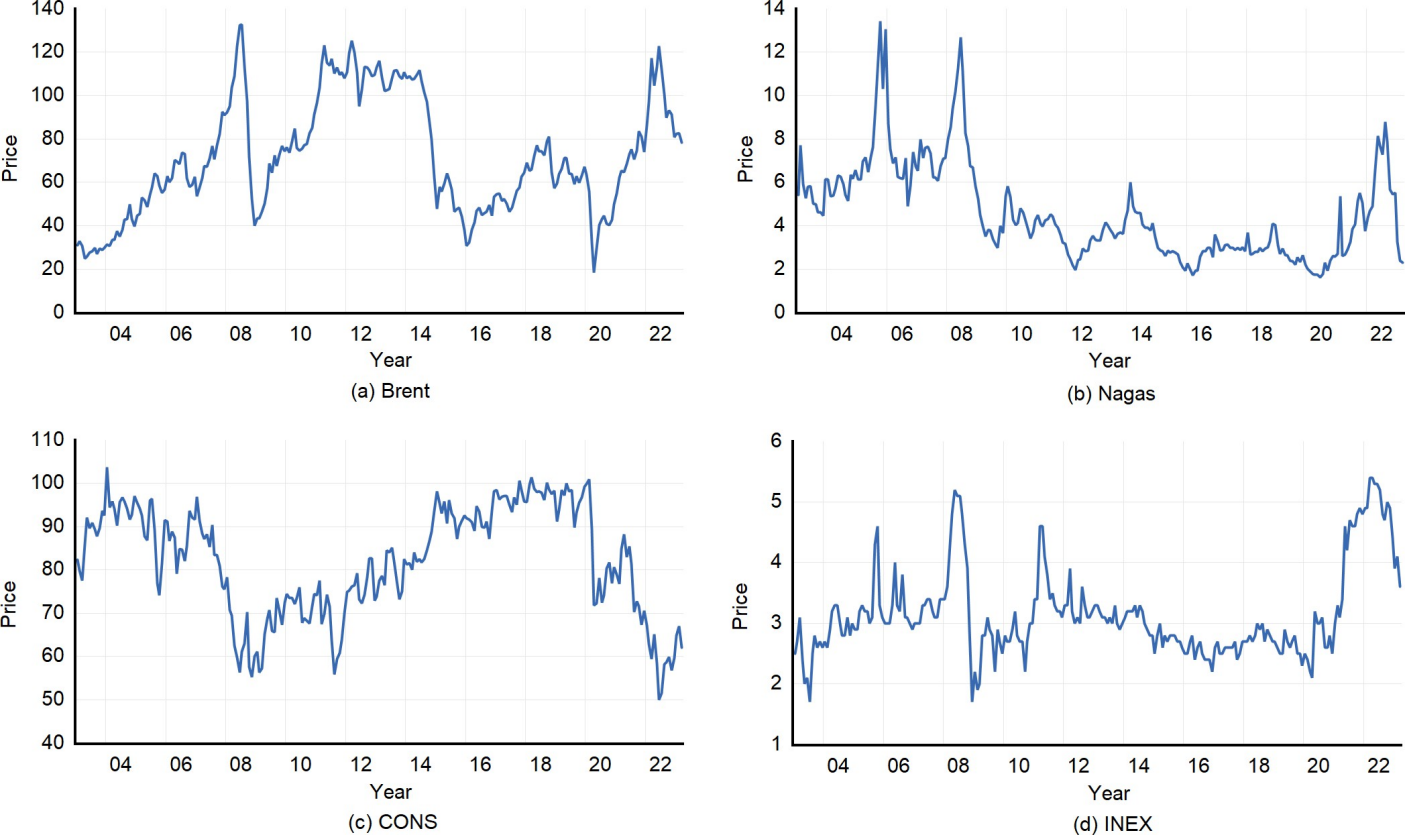
Trends of four variables.

Fig 1 indicates the fluctuations of the four time series throughout the sample period. The Brent crude oil price has exhibited significant volatility since 2003, with a notable surge in 2022 that nearly reached its all-time high recorded in early 2008. In contrast, the natural gas price has demonstrated relative stability, generally oscillating within the range of $2-$6 per million British thermal units (MMBtu) for most periods. CONS and INEX both underwent substantial fluctuations during the sample interval and signaled an inverse trend. Specifically, CONS declined between 2006 and 2008 and reached a 20-year low around 2022, while INEX approached an all-time high from 2006 to 2008 and reached a 20-year high around 2022.

Table 1 summarizes the descriptive statistics of all variables. Brent crude oil and CONS show the greatest volatility, with near-zero skewness, indicating a near-normal distribution. Natural gas and INEX have more stable volatility, positive skewness, and kurtosis above 3, presenting distributions with sharp peaks and thick tails.

**Table 1:** Descriptive statistics.

Variables	Mean	Median	Maximum	Minimum	Std. Dev	Skewness	Kurtosis
Brent	71.43	67.31	132.72	18.38	27.02	0.30	2.10
Nagas	4.59	4.01	13.42	1.63	2.23	1.31	4.96
CONS	82.14	83.40	103.80	50.00	12.69	-0.43	2.17
INEX	3.15	3.00	5.40	1.70	0.75	1.29	4.27

The results of unit root tests for each variable are demonstrated in Table 2. The Augmented Dickey-Fuller (ADF) and Phillips-Perron (PP) tests reveal that the first-order difference series of the four variables are stationary. To account for potential structural breaks often omitted in standard tests, the Zivot-Andrews (ZA) test [56] is employed. In this test, the null hypothesis of the existence of a unit root is rejected at the 0.1% significance level for all variables, confirming their stationarity with structural breaks. Notably, the timing of these breakpoints corresponds to major events such as the global financial crisis in 2008, the European sovereign debt crisis in 2011, the oil price crisis and stock market crash around 2015, and COVID-19 in 2020 [57, 58].

**Table 2:** Unit root tests.

Variables	ADF-Test		PP-Test		ZA-Test		
	I (0)	I (1)	I (0)	I (1)	T-statistic	k	Breakpoint
Brent	-2.88**[1]	-10.92***[0]	-2.58*[3]	-10.79***[5]	-5.06***	1	2014M07
Nagas	-2.82*[0]	-16.95***[0]	-2.91**[6]	-16.88***[4]	-5.05***	0	2008M08
CONS	-1.91[2]	-13.71***[1]	-2.01 [7]	-16.36***[14]	-3.28**	2	2013M11
INEX	-3.53***[0]	-16.09***[0]	-3.54***[1]	-16.26***[7]	-4.49***	4	2020M03

Notes: The optimal order is chosen based on the AIC quasi-test; for the ADF test, the number in [] denotes the optimal lag; for the PP test, [] denotes the bandwidth chosen using the Bartlett kernel, and k in the ZA test denotes the optimal lag order chosen. *, ** and *** denote significance levels of 10%, 5%, and 1%, respectively.

In summary, after the descriptive statistical analysis and the unit root test, the data is deemed suitable for proceeding with wavelet analysis and NARDL model test.

### 4.2. Wavelet analysis

**Fig 2:**
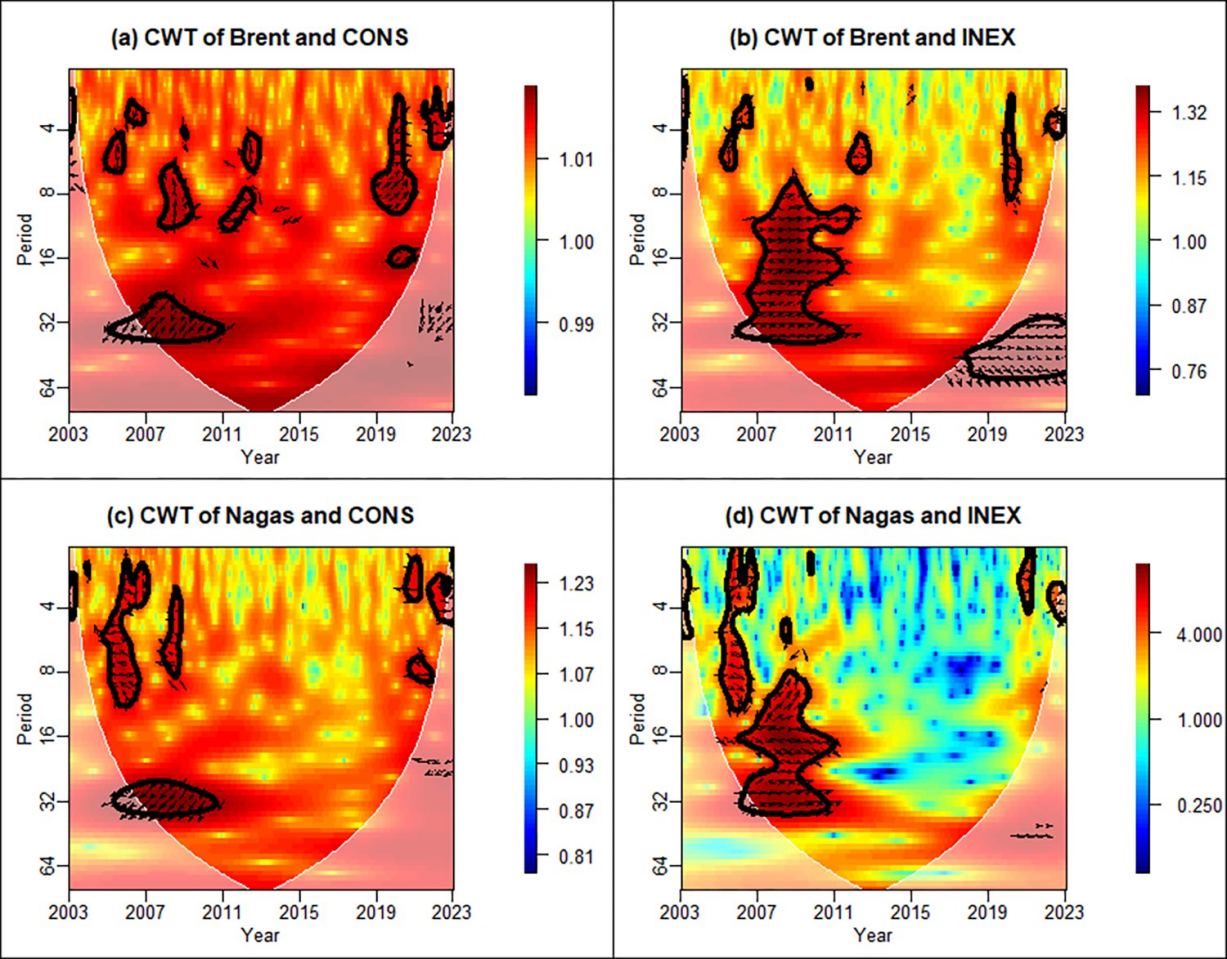
Cross-wavelet transforms between CONS, INEX, and energy prices. Note: In cross-wavelet mapping, phase (lag-lead) relationships are shown by the arrows. Specifically, arrow → (←) indicates that the two sequences are positively (negatively) correlated; arrow ↑ (↓) indicates that the leading indicator (energy price) is ahead of energy price (leading indicator). The arrow ↗(↘) indicates that the series are in-phase, but the leading indicator (energy price) is the dominant factor influencing the energy price (leading indicator); conversely, arrow ↖(↙) indicates that the series are out-of-phase, but the energy price (leading indicator) is the dominant factor causing the leading indicator (energy price).

The cross-wavelet transform is a sophisticated analytical tool that assesses not only the degree of interrelationships between signals but also their phase relationships within the time-frequency domain. Fig 2 reveals the results of the cross-wavelet transform between Brent crude oil, natural gas, CONS and INEX. The plot elements are interpreted as follows: (1) The x-axis denotes the time components, spanning from 2003 to 2013.The y-axis depicts the frequency domain components of the period from 4 to 64. It is essential to note that long wavelet periods correspond to low-frequency movements, whereas short wavelet periods correspond to high-frequency movements. The thick white contours indicate the 95% significance level, and the white lines are influence cones. (2) The warmth or coldness of the colors represent the strength of the co-movement between the two sequences, with warmer colors (red) representing strong synergistic movements and colder colors (blue) indicating weaker synergistic movements. (3) Arrows represent phase relationships. For the convenience of subsequent research and interpretation, as far as the research object of this paper is concerned, we set the energy price in the first serial position(y), and the two leading indicators of CONS and INEX in the second serial position(x).

Fig 2(A) demonstrates the cross-wavelet transform between Brent crude oil price and CONS with strong overall synergies. As the leading factor, CONS exhibits negative long-term(low-frequency) co-movements during 2007–2011, while the mid and high-frequency plots irregular leading and lagging movements. During 2019–2021, the correlation displays a continuous positive synergy. This finding aligns with Zhao’s research [15], suggesting that online investor sentiment can strengthen the correlation between oil prices and external events, especially under the ravages of the COVID-19 epidemic. Fig 2(B) displays a positive co-movement between Brent oil price and INEX in the medium and long runs from 2007 to 2011, and INEX as the leading factor.

Fig 2(C) mirrors the continuous long-run(low-frequency) negative synergy during 2007–2011 like Fig 2(A), when CONS is the dominant factor influencing natural gas prices. In contrast, during 2004–2006, short-run negative synergy is led by natural gas. Fig 2(D) shows that, contrary to Fig 2(C), the positive synergies in the short and medium runs during 2005–2006, are still evident and dominated by natural gas, while a sustained positive co-movement from 2006 to 2011 is dominated by INEX.

In summary, the co-movements between leading indicators and the energy prices are mainly concentrated in the 2006–2011. The relationship between CONS and energy price exhibits a long-term negative movement dominated by CONS. While INEX dominates the medium and long-term positive movement of energy prices. Compared to the long-run variance, the temporal distribution of the low and medium-run movements is more dispersed, with high-frequency correlations between energy prices and both CONS and INEX around 2020, potentially linked to the recurrence of the COVID-19 epidemic. Long et al. [59] exploited continuous wavelet to identify a positive correlation in the medium and high-frequency domains, corroborating our findings.

**Fig 3:**
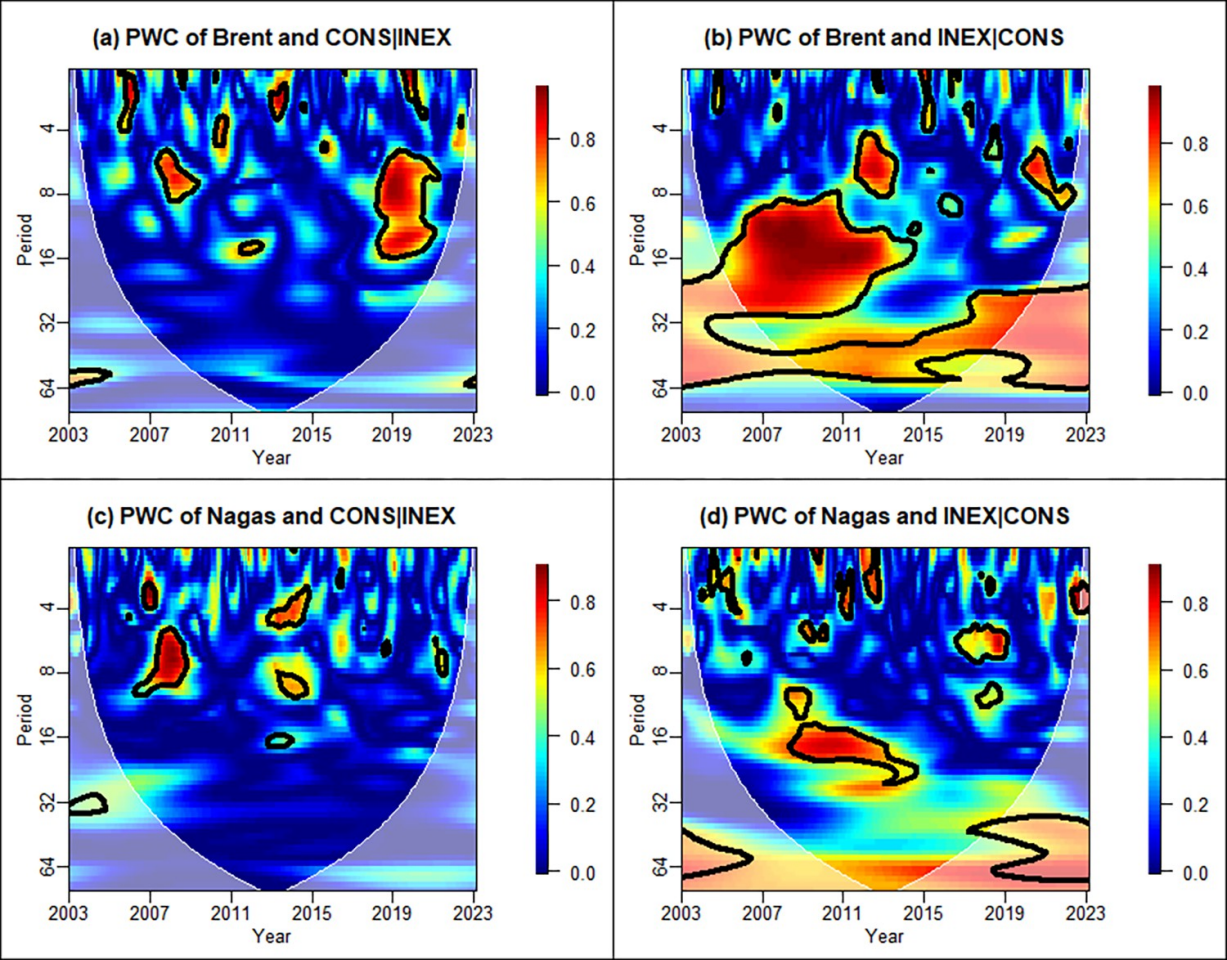
Partial wavelet coherence between CONS, INEX, and energy prices.

To eliminate the interference caused by the third variable, we employ the partial wavelet coherence test, as illustrated in Fig 3. Defining energy prices as the dependent variable while separately defining CONS and INEX as independent variables eliminates the effect of the other.

In Fig 3(A), there exists a certain degree of short-term correlation between Brent crude oil and CONS in 4 to 8 bands during 2007–2011 when eliminating the effect of INEX on it, while the correlation is more noteworthy in the mid-frequency bands during 2018–2021. When excluding the interference of CONS, there exists noticeable medium and long-term coherence in the 8–32 band during 2007–2013. Mensi et al. [60] similarly confirmed the evident medium-run relationship between oil changes and inflation in several countries during the 2008 Global Financial Crisis (GFC) and the 2012 European Sovereign Debt Crisis (ESDC). This aligns with our findings well.

Similarly, the correlation between CONS and natural gas price demonstrated in Fig 3(C) is similar to Fig 3(A), but the effect is not significant enough. Fig 3(D) depicts that the correlation between INEX and natural gas prices is more apparent in the medium-term during 2008–2012.

**Fig 4:**
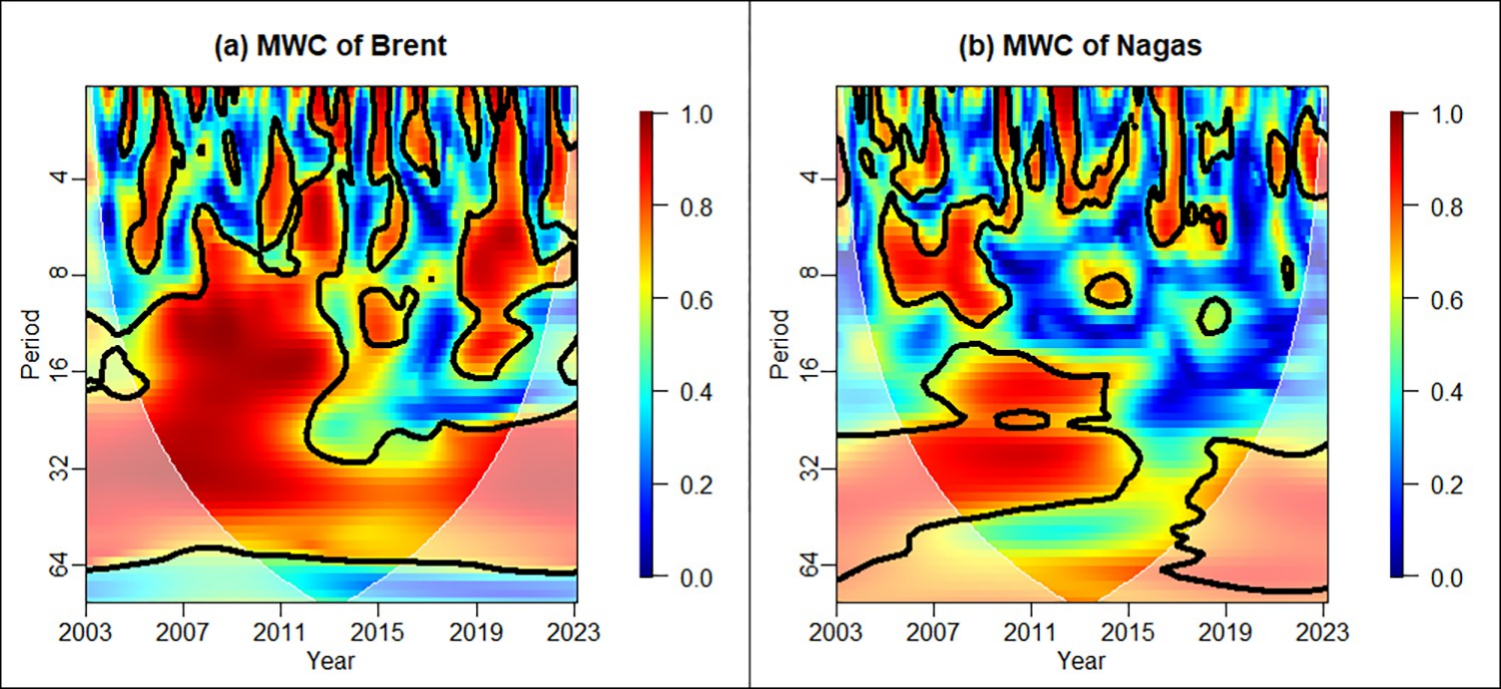
Multiple wavelet coherence between CONS, INEX, and energy prices.

Multiple wavelet coherence allows for the cumulative effect of multiple independent variables on the dependent variable. By comparing the outcomes of multiple versus single wavelet coherence analyses, we can more effectively discern the distinct influences of various factors on energy prices across different time-frequency bands. To examine the joint impact of CONS and INEX on the energy price, Fig 4 signals clear correlations in all periods. Notably, the significance level of the multiple wavelet coherence extends across nearly the entire cone of influence, with a heightened presence in the high-frequency band compared to the wavelet coherence analysis with a single independent variable.

Upon synthesizing the findings from both wavelet coherence methods, we conclude the following: Firstly, there exist significant linkages between CONS, INEX, and international energy prices, with Brent crude oil showing stronger linkages than natural gas, especially during the financial crisis in 2008 and the outbreak of COVID-19 in 2019–2021. Secondly, the correlation between CONS and energy prices is predominantly short-term, whereas the correlation between INEX and energy prices is mainly in the medium and long-terms. Overall, the linkage between INEX and energy prices is more pronounced. Since the fall of 2021, inflation expectations have surged sharply from their lowest levels at the end of 2020. The expected rapid recovery will result in higher inflation, while an increase in policy interest rates will amplify the impact of positive shocks to long-term inflation expectations on real inflation and economic activity [61].

### 4.3. Results of NARDL test

Wavelet analysis explores the correlation between the series in terms of time and frequency but does not account for the potential asymmetric effects of independent variable on the dependent variable. To address this, we proceed with NARDL for subsequent tests. Models 1 and 2 are formulated with Brent crude oil price and natural gas price as respective explanatory variables, while CONS and INEX are disentangled into positive and negative changes as independent variables.

To evaluate the cointegration relationship among the variables, the bound test is performed in Table 3. First, the F-statistic of model 1 is 3.95, which is much larger than the non-stationary boundary of I (1) at the 5% significance level, indicating that the original hypothesis of no cointegration is rejected. In parallel, the F-statistic of model 2 is 7.24, which confirms the existence of a cointegration relationship.

**Table 3:** Bound tests.

Model	F-statistic	5%		1%	
		I (0)	I (1)	I (0)	I (1)
Model 1: Brent, CONS^+^, CON^-^, INEX^+^, INEX^-^	3.95	2.56	3.49	3.29	4.37
Model 2: Nagas, CONS^+^, CON^-^, INEX^+^, INEX^-^	7.24	2.56	3.49	3.29	4.37

Note: I (0) and I (1) are smooth and non-smooth boundaries, respectively.

After verifying the cointegration relationship, the results of NARDL estimation are observed in Table 4. In Model 1, the positive short-term coefficient of CONS is 0.34, with a negative coefficient is 0.43. For Inflation Expectations, the positive short-term coefficient is 6.15 and the negative coefficient is 9.19. All these estimates reached the significance level of 5% or higher. The results indicate that, at the short-run level, positive shocks of CONS are negatively associated with crude oil price and negative shocks are positively associated with crude oil price, and there is a remarkable asymmetry between the two shocks. Specifically, a 1% increase in CONS results in a 0.34% decrease in crude oil prices, while a 1% decrease in CONS also makes for a 0.43% decrease. Both positive and negative shocks to INEX are negatively correlated with the crude oil price, with negative shocks (9.19%) having a more substantial effect than positive shocks, indicating a significant asymmetric influence with reverse shocks being predominant.

**Table 4:** NARDL estimation.

Variables	Model1(2,0,1,1,1)			Variables	Model2(1,0,0,0,0,3)		
	Brent				Nagas		
	Coeff.	T-stat	Std.		Coeff.	T-stat	Std.
Long-term estimation							
Constant	6.44***	4.22	1.53	Constant	2.20***	5.54	0.40
CONS^+^ (-1)	-0.14***	-2.99	0.05	CONS^+^	0.01**	1.87	0.01
CONS^-^ (-1)	-0.02	-0.50	0.05	CONS^-^	-0.01	-0.79	0.01
INEX^+^ (-1)	2.75**	2.35	1.17	INEX^+^ (-1)	0.37***	3.19	0.12
INEX^-^ (-1)	1.01	1.44	0.70	INEX^-^ (-1)	0.65***	5.31	0.12
Short-term estimation							
Δ CONS^+^	-0.34**	-1.91	0.18	Δ INEX^+^	0.33	1.37	0.24
Δ CONS^-^	0.43***	2.92	0.15	Δ INEX^-^	1.21***	4.41	0.27
Δ INEX^+^	6.15***	3.23	1.90	Δ INEX^+^ (-1)	0.31	1.28	0.24
Δ INEX^-^	9.19***	4.19	2.19	Δ INEX^-^ (-1)	-0.76***	-2.79	0.27
Long-term coefficients							
LR CONS^+^	-1.38***	-3.73	0.37	LR CONS^+^	0.04**	1.88	0.02
LR CONS^-^	-0.23	-0.49	0.47	LR CONS^-^	0.01	0.44	0.03
LR INEX^+^	26.68***	3.30	8.09	LR INEX^+^	1.48***	3.32	0.45
LR INEX^-^	9.20	1.49	6.16	LR INEX^-^	2.10***	6.14	0.34

Notes: *, **, *** indicate statistical significance at the 10%, 5% and 1% level. + and–show positive and negative changes in energy prices. (-1) shows dependent variables on lag (1).

Compared to the short-run results, the NARDL test for the long-run level of Model 1 is more complicated. First, the positive shocks in the long-run shocks of CONS and INEX on oil price reach the significance levels of 1% and 5%, respectively, while the effect of negative shocks is not noteworthy. The long-run coefficient estimates imply that positive shocks to CONS are negatively associated with crude oil price, with oil price falling by 1.38% for every 1% increase in CONS. Conversely, positive shocks to INEX are positive, with crude oil price increasing by 26.68% for every 1% rise in INEX.

The results for natural gas differ evidently from those for crude oil as shown in Model 2. In the short-run, the impact of CONS on natural gas prices is statistically insignificant, suggesting no short-run shocks. However, the negative shock of INEX is significant, with the inverse shock of the original series positively correlating with natural gas price. Yet, the inverse shock of the first order lagged INEX is negatively correlated with natural gas prices. Normally, the onset of inflation gives rise to price increases; however, INEX is an anticipation of the occurrence of inflation, and measures to guard against inflation in turn trigger asset prices to rise, i.e., the expectation of inflation itself accelerates the onset of inflation. The results of the estimation at the long-term level illustrate that the long-term impact of INEX on natural gas price remains significant and reveals a positive correlation, with a 1% increase in positive shocks to INEX increasing natural gas prices by 1.48%, and a 1% increase in negative shocks lowering it by 2.10%, overall indicating that negative shocks have a more dominant role. On the other hand, CONS appears to have little impact on natural gas prices, suggesting that its impact is not demonstrable, making the demonstration of asymmetry more challenging.

The results of NARDL illustrate the following: there are short-run asymmetric shocks to crude oil price from CONS and INEX, and the reverse shock dominates; in the long-run, the positive shocks from both factors are the primary drivers of asymmetry for oil prices, while the reverse shocks are not significant. For natural gas, INEX exerts asymmetric effects in short and long terms, and both are dominated by reverse shocks, while the asymmetric effect of CONS on natural gas prices is comparatively weak. In this regard, Orlowski et al. [62] confirm that there is a complex interaction between INEX and various commodity futures prices, with crude oil futures prices, in particular, responding strongly and positively to changes in INEX.

Table 5 presents the overall results of asymmetry tests, which are consistent with the results of the parameter estimates above. Collectively, there exists a significant asymmetric effect of CONS and INEX on the price of Brent crude oil; the asymmetric effect of INEX on the price of natural gas has also been confirmed, whereas the impact of CONS on natural gas does not reach statistical verification.

**Table 5:** Asymmetric long-run and short-run tests.

Variables	Model1		Model2	
	F-statistic	Chi-square	F-statistic	Chi-square
Long-term coefficients				
CONS	5.01**	5.01**	7.08**	7.08**
INEX	4.95**	4.95**	10.55***	10.55***
Short-term coefficients				
CONS	8.02***	8.02***	**——**	**——**
INEX	0.90	0.90	0.14	0.14
Total coefficients				
CONS	6.14***	12.28***	**——**	**——**
INEX	3.26**	6.51**	5.72***	11.45***

Note: *, **, *** indicate statistical significance at the 10%, 5% and 1% level.

**Fig 5:**
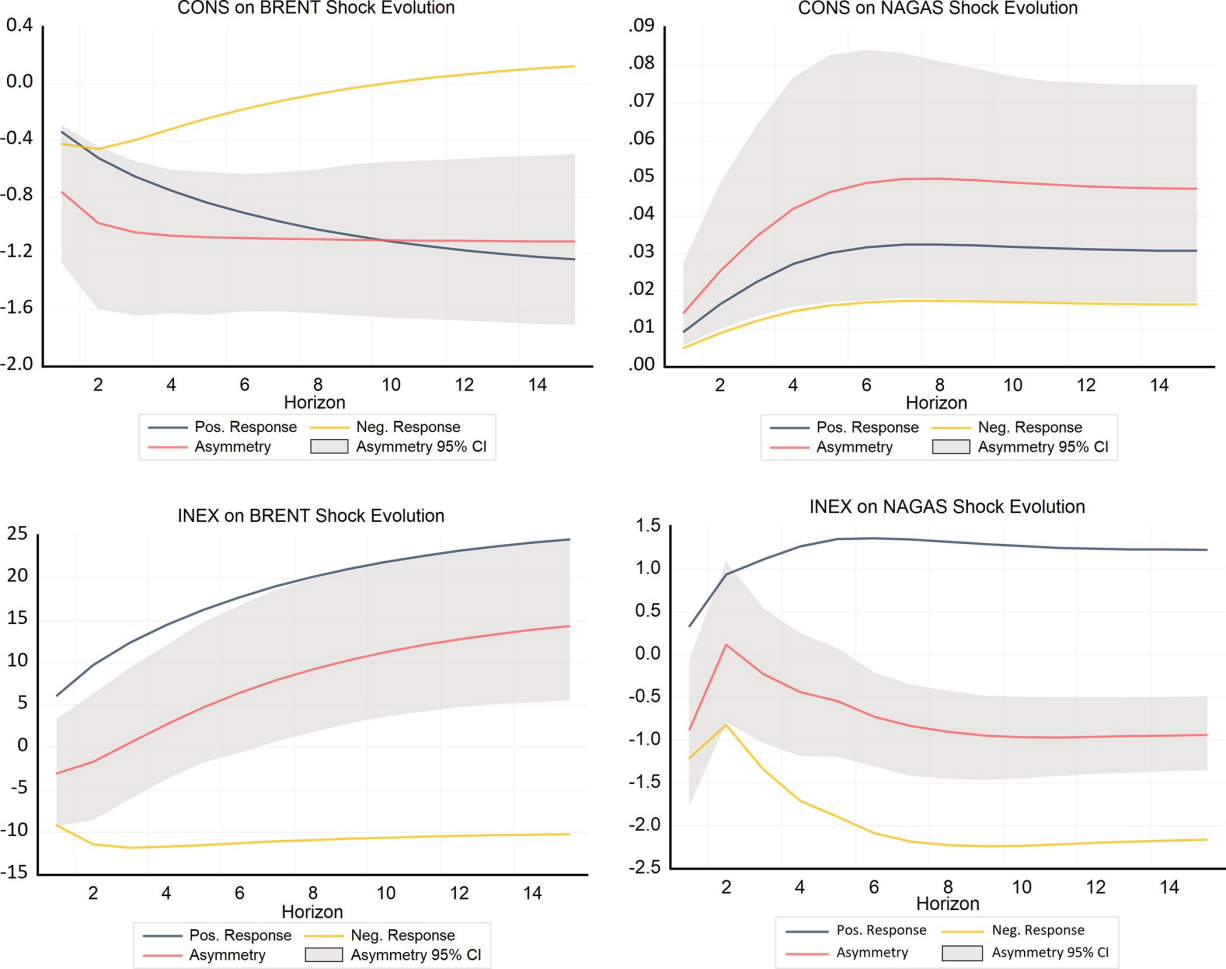
Asymmetric dynamic cumulative multipliers.

To visualize the asymmetric shocks of CONS and INEX on energy prices, the results of the above study are presented using asymmetric dynamic cumulative multipliers. In Fig 5, the cumulative dynamic nonlinear multiplier results are up to 15 months long. The blue and yellow lines in the figure indicate positive and negative shocks to energy prices, respectively, while the red line represents the asymmetric combined effect of positive and negative shocks on energy prices, and the grey background indicates the 95% critical boundary.

Fig 5(A) indicates the asymmetric dynamic shocks of CONS on crude oil price. In accordance with the previous results, positive shocks are negatively correlated with changes in crude oil price, and negative shocks are positively correlated with crude oil price, together manifesting significant reverse asymmetry. Fig 5(B) exhibits the asymmetric shocks of INEX on crude oil price and in the short run the negative shocks dominate. Whereas in the long run, the negative shocks stabilize at around 10%, the positive shocks gradually increase and dominate to reach the level of 25%. This is in line with the finding by Ascari et al. [63] that shocks increasing INEX have a more substantial effect on real variables compared to shocks that decrease INEX, reflecting an asymmetric transmission of inflation expectations shocks.

Similarly, Fig 5(C) reveals the asymmetric dynamic shocks of INEX on natural gas prices. Although both positive and negative shocks are positively correlated with natural gas price, the coefficients are less than 0.1, indicating that asymmetry is not pronounced. Fig 5(D) further reveals the asymmetric impact of INEX on natural gas prices, with negative shocks occupying a relatively dominant position in both the short and long run. Thus, all the results of the dynamic multiplier test corroborate the above NARDL asymmetric test results.

**Fig 6:**
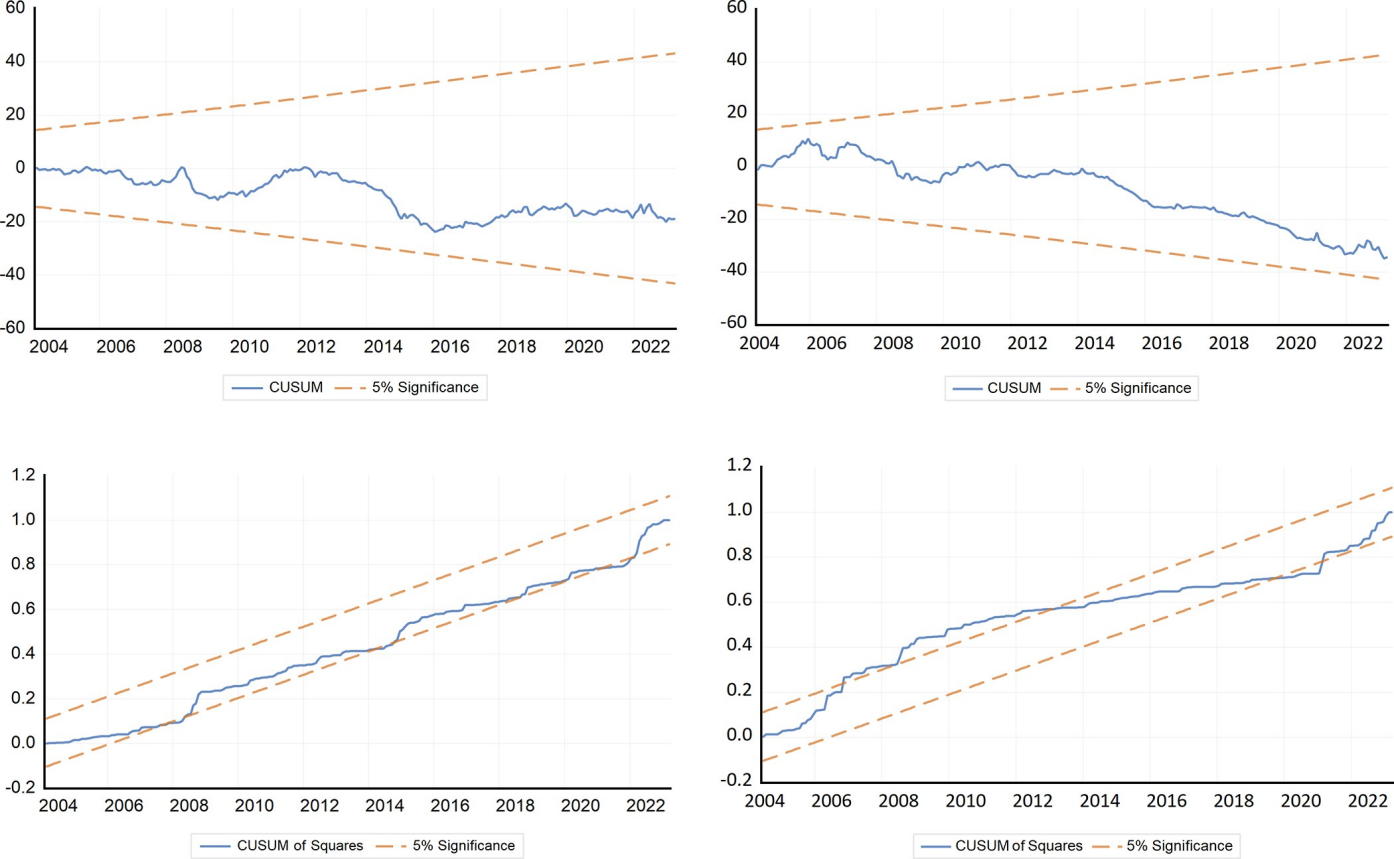
CUSUM and CUSUMQ tests.

Finally, we employed the CUSUM and CUSUMQ techniques to assess the stability of the two models at the 5% significance level. The results, as demonstrated in Fig 6 indicate that both models, featuring Brent crude oil and natural gas as explanatory variables have passed the stability test.

We compare the above results with past empirical evidence and find most have yielded similar results to ours. Jawadi et al. [64] proved that extra-financial news about investment sentiment will drive the volatility of energy prices and the effect tends to be nonlinearity and threshold. Specifically, there exist different forms of lead-lag effects between investor sentiment and energy prices and the former is a significant driver of the latter. The study of Huang [21] about gold price indicates that both market sentiment and inflation expectations significantly influence gold price when their levels are beyond a certain threshold, but the latter lasts longer. In addition, some investigations implied that when inflation expectations increase, the reaction of prices to exchange rates intensifies [18]. Therefore, effective management of inflation expectations can help stabilize exchange rate fluctuations. It is also worth noting that Berger et al. [65] found that after the outbreak of a financial crisis in 2008, dependence schemes between S&P 500 and commodity become stronger, with a higher likelihood of joint extreme movements and a greater degree of time variance. Currently, research into the impact of external factors on price asymmetry is scarce, yet numerous scholars have documented the asymmetric effects of oil price shocks on macroeconomics, offering valuable insights for our findings. For example, Jawadi et al. [66] employed a threshold model to demonstrate the substantial influence of oil price shocks on inflation. Moreover, the research conducted by Bossman et al. [67] further verified the asymmetric relationship between market sentiment and stock prices. Consequently, by comparing with previous studies, we believe that the empirical results of this paper are reasonable to a certain extent.

### 4.4. Robustness test

To substantiate the robustness of our findings, we selected the spot price of WTI crude oil as the explanatory variable, employing the same sample period and variable set for the estimation of the NARDL model in Table 6. The analysis yields the following insights: First, in the short-term, there is a conspicuous asymmetric effect of both CONS and INEX on energy prices, with reverse shocks being predominant. Second, in the face of both positive and negative shocks to inflation expectations, the response of WTI crude oil price is positively correlated with them. Third, in the long run, the positive shock of CONS on the WTI oil price reaches a significant level, while the negative shock does not reach significance. For INEX, both the positive and negative shocks of INEX on the WTI oil price reach significance at the levels of 5% and 10%, respectively, with the positive shock being more influential.

**Table 6:** Robustness tests.

Variables	WTI		
	Coeff.	T-stat	Std.
Long-term estimation			
Constant	6.92***	4.35	1.59
CONS^+^ (-1)	-0.12***	-2.75	0.04
CONS^-^ (-1)	-0.03	-0.80	0.04
INEX^+^ (-1)	2.38**	2.26	1.05
INEX^-^ (-1)	1.18*	1.74	0.68
Short-term estimation			
Δ CONS^+^	-0.26	-1.56	0.17
Δ CONS^-^	0.42***	3.08	0.14
Δ INEX^+^	6.11***	3.44	1.77
Δ INEX^-^	8.40***	4.02	2.09
Long-term coefficients			
LR CONS^+^	-1.09***	-3.25	0.33
LR CONS^-^	-0.33	-0.77	0.42
LR INEX^+^	22.40***	3.05	7.35
LR INEX^-^	11.13**	1.97	5.64

Note: *, **, *** indicate statistical significance at the 10%, 5% and 1% level.

Comparing the above results with the NARDL estimation of Brent crude oil as the explanatory variable, we find that the short-term shocks of CONS and INEX on both types of oil price are markedly asymmetric, with negative shocks being dominant. Moreover, the positive shocks of CONS and INEX on both types of crude oil price are significant in the long run, with INEX showing a particularly strong effect. In conclusion, the application of the NARDL model to investigate the asymmetric effect of CONS and INEX on international energy prices has proven to be robust, demonstrating consistent results across different oil benchmarks.

## 5. Conclusion and implications

This paper explores the dynamic linkage and asymmetric effects of consumer sentiment, inflation expectations, and international energy prices, which is a new investigation based on the perspective of behavioral economics. Brent crude oil and natural gas prices serve as proxies for international energy prices, with sample intervals ranging from January 2003 to March 2023.

First, we employ three wavelet techniques to plumb the dynamic co-movements between consumer sentiment, inflation expectations and international energy prices from a time-frequency perspective. The results of cross-wavelet display that there are medium and long-run synergies between these variables during 2006–2011. Notably, the relationship between consumer sentiment and energy prices is primarily negative, while that between inflation expectations and energy prices is positive. In contrast to the long-run co-movement, the medium and short-run correlations is more sporadic, with a notable surge around 2020. Partial and multiple wavelet coherence analysis indicate that the correlation between consumer sentiment and energy prices mainly in the high-frequency band(short-term), whereas the correlation between inflation expectations and energy prices is more evident in the medium and long-term. Overall, the correlation between inflation expectations and energy prices is more pronounced.

Second, a NARDL model is utilized to investigate the asymmetric effects of consumer sentiment and inflation expectations on crude oil and natural gas prices, respectively. Firstly, the findings indicate short-term asymmetric shocks on Brent crude oil prices from both variables, with reverse shocks being more pronounced. In the long term, the positive shocks from both factors are the main drivers of asymmetry for oil prices. Secondly, inflation expectations exert short and long-run asymmetric effects on natural gas prices, characterized by reverse shocks, while consumer sentiment has a weak effect on natural gas prices and the asymmetric effect is not noticeable.

The above findings give us quite a few policy implications. The volatility of international energy prices is affected by consumer sentiment and inflation expectations, necessitating comprehensive management of these factors by governments and regulatory bodies to maintain stable and healthy market conditions.

1. Consumer sentiment often affects the subsequent consumption behavior of consumers, the increase or decrease in consumption will inevitably affect the price, thus affecting the stability of the market. In this regard, the relevant departments should construct and track sentiment indices to make judgments timely and take necessary measures to mitigate risks and bolster consumer confidence. For instance, enhancing consumer trust through transparent communication is desirable and can further stimulate consumption.
2. Inflation is not only a monetary but also an obvious institutional phenomenon. Government fiscal spending will increase the upward pressure on global inflation in a short period, and although this effect will diminish in the long term, it should not be ignored [68]. A decline in long-term inflation expectations will lead to a contraction in the economic. Policymakers should encourage industrial production and increase interest rates in the short term. Long-term strategies should focus on eliminating systemic inflationary factors, strengthening the coordination and mutual constraints between important macro policies, and enhancing the endogenous nature of economic development. Close monitoring of financing growth and refining macroeconomic regulatory mechanisms are also essential. For both businesses and investors, when facing inflation shocks, it is necessary to adjust investment strategies and optimize investment portfolios promptly.
3. Under the impact of external events such as financial crises and pandemic outbreaks, the relationship between consumer sentiment, inflation expectations, and energy prices may become increasingly unstable. Incomplete dissemination of public opinion information will result in information asymmetry, thereby exacerbating the negative psychology and panic buying of the public. Government departments should first make appropriate use of social media platforms to release positive information, alleviate market panic caused by crisis events, and implement stimulus plans to disseminate consumer confidence and stabilize market development. At the same time, effective rewards and publishments need to be established to prevent false announcements and rumors. In addition, it is even more necessary to increase capital investment, accelerate infrastructure construction, and enhance the market’s ability to resist risks.
